# Meeting report: Convening on the influenza human viral challenge model for universal influenza vaccines, Part 1: Value; challenge virus selection; regulatory, industry and ethical considerations; increasing standardization, access and capacity

**DOI:** 10.1016/j.vaccine.2019.06.080

**Published:** 2019-08-14

**Authors:** Bruce L. Innis, Francesco Berlanda Scorza, Jeremy S. Blum, Varsha K. Jain, Anastazia Older Aguilar, Diane J. Post, Paul C. Roberts, Niteen Wairagkar, Janet White, Joseph Bresee

**Affiliations:** a Center for Vaccine Innovation and Access, PATH, 455 Massachusetts Avenue NW, Suite 1000, Washington, DC 20001, USA; b Bill & Melinda Gates Foundation, PO Box 23350, Seattle, WA 98102, USA; c Division of Microbiology and Infectious Diseases, National Institute of Allergy and Infectious Diseases, National Institutes of Health, Bethesda, MD 20892-9826, USA; d Influenza Division, Centers for Disease Control and Prevention, and Task Force for Global Health, Atlanta, USA

**Keywords:** Influenza, Influenza vaccine, Universal vaccine, Human challenge trial, Vaccine development

## Abstract

In response to global interest in the development of a universal influenza vaccine, the Bill & Melinda Gates Foundation, PATH, and the Global Funders Consortium for Universal Influenza Vaccine Development convened a meeting of experts (London, UK, May 2018) to assess the role of a standardized controlled human influenza virus infection model (CHIVIM) towards the development of novel influenza vaccine candidates. This report (in two parts) summarizes those discussions and offers consensus recommendations. This article (Part 1) covers challenge virus selection, regulatory and ethical considerations, and issues concerning standardization, access, and capacity. Part 2 covers specific methodologic considerations.

Current methods for influenza vaccine development and licensure require large costly field trials. The CHIVIM requires fewer subjects and the controlled setting allows for better understanding of influenza transmission and host immunogenicity. The CHIVIM can be used to identify immune predictors of disease for at-risk populations and to measure efficacy of potential vaccines for further development.

Limitations to the CHIVIM include lack of standardization, limited access to challenge viruses and assays, lack of consensus regarding role of the CHIVIM in vaccine development pathway, and concerns regarding risk to study participants and community. To address these issues, the panel of experts recommended that WHO and other key stakeholders provide guidance on standardization, challenge virus selection, and risk management. A common repository of well-characterized challenge viruses, harmonized protocols, and standardized assays should be made available to researchers. A network of research institutions performing CHIVIM trials should be created, and more study sites are needed to increase capacity.

Experts agreed that a research network of institutions working with a standardized CHIVIM could contribute important data to support more rapid development and licensure of novel vaccines capable of providing long-lasting protection against seasonal and pandemic influenza strains.

## Introduction

Influenza is an ongoing public health challenge, causing significant worldwide morbidity and mortality. There is increasing global interest in the development of a universal vaccine that would provide broad long-lasting protection against both seasonal and pandemic influenza.

Current seasonal influenza vaccines target specific virus strains by inducing antibodies to the head region of the viral hemagglutinin protein (HA). This region undergoes gradual antigenic changes over time - antigenic drift - which can reduce the efficacy of seasonal vaccines and requires updating vaccines semiannually to match evolving circulating strains. Due to their strain-specific design, current seasonal vaccines are less effective against novel or pandemic strains. A broadly protective “universal” vaccine would be capable of inducing immunity to multiple influenza type A and B viruses and would provide longer lasting protection against both seasonal and pandemic influenza.

Research and technologic advances have resulted in the development of innovative vaccine strategies based on better understanding of the pathogenesis of influenza and host immunologic responses [1]. However, current methods for influenza vaccine development and licensure require large costly field trials to demonstrate vaccine efficacy in prevention of illness or in attaining an immune response predictive of clinical benefit or non-inferior to that elicited by an already approved vaccine of the same class. These trials rely on traditional immunological endpoints such as hemagglutination inhibition (HI) antibody titers, which do not reflect non-neutralizing immune response mechanisms. Novel vaccines intended to elicit broad immunity to conserved influenza virus antigens can only be evaluated for acceptable immunogenicity if new immunologic endpoints correlated with clinical benefit are defined and validated. Animal models can contribute useful information but do not fully represent the human disease response. There is a need for better correlates of protection (CoPs) [2] (markers of immune function that statistically correlate with protection after vaccination) for populations at-risk for severe disease (infants, pregnant women, and the elderly).

Human challenge trials (HCTs) may be a useful additional tool for development and evaluation of novel universal vaccines. HCTs require fewer participants and can be used to assess efficacy for more rapid selection of promising vaccine candidates. Due to their controlled nature, challenge trials can contribute to a detailed understanding of how immune responses to a vaccine candidate modify the outcomes of an exposure to a viral inoculum in terms of virus replication and symptoms of illness; this could define new CoPs. Current human influenza challenge models have some limitations, however. Certain artificial characteristics of current models (such as viral load and route of administration) prevent them from adequately representing community-acquired infections (CAIs). (See “Route of Administration and Dose” in the companion article about CHIVIM methodology) [3]. There is a limited set of influenza viruses available for challenge, a lack of standardization among models, and a limited number of research centers that have experience in conducting influenza challenge trials.

The Bill & Melinda Gates Foundation (BMGF), PATH and the Global Funders Consortium for Universal Influenza Vaccine Development convened a meeting of approximately 60 experts (Table 1) in London, UK (May 31- June 1, 2018) to collect perspectives and identify best practices on how to improve the utility of a standardized controlled human influenza virus infection model (CHIVIM). Participants included vaccine researchers, public health officials, regulatory experts, and representatives from the pharmaceutical industry. The meeting focused on five topics, each with a set of critical pragmatic questions. Leaders in the field chaired each session and moderated discussion panels to address the questions in depth with audience input. This report (in two parts) summarizes those discussions and offers recommendations to improve CHIVIM as a necessary tool for universal influenza vaccine development.

**Table 1:** Participant List for Convening on the Influenza Human Challenge Model for Universal Influenza Vaccine Development.

**Participants**	
Pedro A. Piedra, MD	Baylor College of Medicine
Asha Abraham, MD, PhD	Christian Medical College, Vellore, Tamil Nadu, India
Stacey Wooden, PhD, MPH	Human Vaccines Project
Adolfo Garcia-Sastre, PhD; Florian Krammer, PhD	Icahn School of Medicine at Mount Sinai
Behazine Combadiere, PhD	Inserm
Claudia Emerson, PhD	McMaster University, Institute on Ethics & Policy for Innovation
Punnee Pitisuttithum, MD, DTMH	Mahidol University, Vaccine Trial Centre, Faculty of Tropical Medicine
Matthew Memoli, MD, MS	NIAID LID Clinical Studies Unit
John Oxford, PhD	Queen Mary College
Mark McKinlay, PhD	Task Force for Global Health, Center for Vaccine Equity
Ab Osterhaus, PhD, DVM	TiHo Hannover Research Center for Emerging Infection & Zoonoses
Eduard Schmidt, PhD	UNISEC Consortium
Rebecca Jane Cox, PhD	University of Bergen Influenza Centre
Donald Milton, MD, DrPH	University of Maryland School of Public Health
Kathleen Neuzil, MD, MPH, FIDSA	University of Maryland Center for Vaccine Development and Global Health
Arnold S. Monto, MD	University of Michigan, School of Public Health
Frederick Hayden, MD	University of Virginia School of Medicine
Yoshihiro Kawaoka, PhD	University of Wisconsin-Madison/University of Tokyo
Shabir Madhi, PhD	University of the Witwatersrand

**Policy**	
Dora Navarro Torne, MD, PhD, PharmD	DG RTD European Commission
Philipp Lambach, MD, MBA, PhD	World Health Organization

**Surveillance and Reference Laboratories**	
Luzhao Feng, MD, PhD	Chinese Center for Disease Control & Prevention
John McCauley, PhD	Francis Crick Institute, WHO Collaborating Centre for Reference and Research on Influenza
Maria Zambon, PhD	Public Health England
Joseph Bresee, MD; David Wentworth, PhD	US Centers for Disease Control & Prevention
Zhiping Ye, MD, PhD	US Food & Drug Administration Laboratory of Respiratory Viruses Diseases

**Regulatory**	
Marco Cavaleri, PhD	European Medicine Agency Anti-Infectives and Vaccines Division
Othmar Engelhardt, Dr nat techn	National Institute for Biological Standards and Control
Jerry Weir, PhD	US Food & Drug Administration Division of Viral Products
Hana Golding, PhD	US Food & Drug Administration Laboratory of Retrovirus Research, Center for Biologics Evaluation and Research

**Industry/Product Development**	
Lidia Oostvogels, MD; Susanne Rauch, PhD	CureVac AG
Marie Van Der Wielen, MD	GSK Vaccines
Andrew Catchpole, DPhil	hVivo
Vivek Shinde, MD, MPH	Novavax, Inc.
Bruce Innis, MD; Francesco Berlanda Scorza, PhD; Gene Saxon, MEng, MME	PATH
Saranya Sridhar, MD, PhD, MPH	Sanofi Pasteur
Jonathan Edelman, MD; Beverly Taylor, PhD	Seqirus, Inc.
Adrian Wildfire, MSc	SGS Life Sciences
John Treanor	Tunnell Government Services
Sean Tucker, PhD	Vaxart, Inc.

**Funders**	
Anastazia Older Aguilar, PhD; Jeremy Blum, PhD; Keith Chirgwin, MD; Varsha Jain, MD; Keith Klugman, MD, PhD; Niteen Wairagkar, MD; Janet White, MBA	Bill & Melinda Gates Foundation
Armen Donabedian, PhD	Biomedical Advanced Research and Development Authority, Flu & EID Vaccines
Marc Ouellette, PhD	Canadian Institutes of Health Research Institute of Infection and Immunity
Casey Wright, MSc	Flu Lab
Emily Erbelding, MD, MPH; Diane Post, PhD; Chris Roberts, PhD	Division of Microbiology and Infectious Diseases, National Institute of Allergy and Infectious Diseases, National Institutes of Health
Shobana Balasingam, MSc; Cecilia Chui, DPhil; Zoe Seager, PhD	Wellcome Trust

This article (Part 1) summarizes the discussion and recommendations from four of the five topics covered at the convening, including realizing the full value of CHIVIM, selection of challenge virus strains, industry and regulatory perspectives, ethical considerations, standardization, and increasing capacity and access. The authors would like to thank the following panelists who shared their expertise: Bruce Innis, MD, PATH; Sean Tucker, PhD, Vaxart, Inc.; Kathleen Neuzil, MD, MPH, FIDSA, University of Maryland Center for Vaccine Development and Global Health; Jerry Weir, PhD, US Food & Drug Administration Division of Viral Products; Marco Cavaleri, PhD, European Medicine Agency Anti-Infectives and Vaccines Division; Diane Post, PhD, Division of Microbiology and Infectious Diseases, National Institute of Allergy and Infectious Diseases, National Institutes of Health; Punnee Pitisuttithum, MD, DTMH, Mahidol University, Vaccine Trial Centre, Faculty of Tropical Medicine; Claudia Emerson, PhD, McMaster University, Institute on Ethics & Policy for Innovation; John Treanor, Tunnell Government Services; Florian Krammer, PhD, Icahn School of Medicine at Mount Sinai; David Wentworth, PhD, US Centers for Disease Control & Prevention; John McCauley, PhD, Francis Crick Institute, WHO Collaborating Centre for Reference and Research on Influenza; Othmar Engelhardt, Dr nat techn, National Institute for Biological Standards and Control; Emily Erbelding, MD, MPH, Division of Microbiology and Infectious Diseases, National Institute of Allergy and Infectious Diseases, National Institutes of Health; Francesco Berlanda Scorza, PhD, PATH; Zoe Seager, PhD, Wellcome Trust; Marie Van Der Wielen, MD, GSK Vaccines; Saranya Sridhar, MD, PhD, MPH, Sanofi Pasteur; Jonathan Edelman, MD, Seqirus, Inc.; and Lidia Oostvogels, MD, CureVac AG. Recommendations reflect a “majority report” with panelists’ recommendations, as reflected in their slides and comments from the dais, and comments from the audience being reported. All panelists and participants had the opportunity to review and comment on a late draft of the meeting report. The companion article “Meeting Report: Convening on the Influenza Human Viral Challenge Model for Universal Influenza Vaccines, Part 2” provides a summary of CHIVIM methods discussed at the meeting, including subject selection and screening, route of exposure and dose, devices for administering challenge, rescue therapy, protection of study participants and institutions, and other methodologic considerations.

## 1 Topic 1: Realizing the full value of CHIVIM

This session focused on exploring the value of human challenge studies as a tool for vaccine development. The panel reviewed the World Health Organization (WHO) Preferred Product Characteristics (PPC) for Next-Generation Vaccines[4] and the BMGF Intervention Target Product Profile[5] for universal influenza vaccines before agreeing on a list of target goals for CHIVIM:

1. The model should measure efficacy of potential vaccines against appropriate outcomes to more efficiently select candidates for field trials.
2. The model should identify surrogates of broad protection as predictors of disease severity in at-risk populations and to optimize dosing
3. The model should help accelerate development of pediatric vaccines
4. The model should be useful for assessing the impact of vaccine candidates on shedding as a surrogate for transmission
5. The model should assess duration of protection.

The CHIVIM requires several key features to achieve these goals. It should induce disease that approximates natural infection in terms of severity, symptoms, and areas of respiratory tract involvement. The challenge virus route of exposure and dose should also simulate CAI. It must be standardized and reproducible, and most importantly, the model should control the risk of harm to study participants and the risk for introduction of novel strains into the community.

### 1.1 Influenza challenge trials, past and present

Early influenza vaccine HCTs in the 1940′s were performed using aerosol inhalation of influenza A or B as the challenge, which induced illness that resembled naturally occurring disease [6], [7]. Though these studies did not meet modern ethical and methodologic standards, vaccine efficacy demonstrated by these early challenge trials was later reproduced in field trials. A key factor for the predictive success of the challenge model was the ability to induce illness via aerosol exposure, as earlier efforts to induce illness by intranasal instillation of virus suspensions were ineffective. These studies also identified the first CoP: lower pre-challenge HI antibody titer was found to be associated with disease. Three decades later, influenza trials returned to intranasal instillation of a liquid viral suspension to administer challenge but added daily assessment of nasopharyngeal washes in cell cultures to quantify virus shedding. This delivery method induced milder symptoms than aerosolized challenge, with disease generally limited to the upper respiratory tract [8], [9], [10]. Although an imperfect model of naturally occurring disease, intranasal delivery of liquid or droplet spray has been the preferred challenge method since that time, as it poses less risk to study participants and significantly lowers risk of community exposure from escaping challenge strains [11].

The largest recent influenza vaccine trial (179 participants) using a human challenge model (NCT02918006) evaluated the efficacy of a novel seasonal influenza virus vaccine candidate compared to a licensed quadrivalent vaccine and placebo. Study participants were screened for baseline HI titers ≤ 10 to ensure sufficient attack rates and received intranasal virus challenge. Despite low levels of pre-challenge immunity, participants experienced insufficient disease to confirm vaccine efficacy for either active treatment, although both vaccines reduced the risk of infection (manifest as viral RNA shedding in nasal swab specimens collected twice per day post-challenge) in a post-hoc analysis. The study did identify individual immune CoPs for each vaccine. The trial was expensive due to the large recruitment requirements (to find participants with low HI titer) and containment costs.

Human challenge models have been used in the development of many other vaccines including cholera and typhoid vaccines [12], [13]. Experts involved in this research shared insights for designing challenge models that support licensure. Optimizing inoculation to achieve the desired attack rates and manifestations of illness (based on natural history of disease) was critical to success, especially in the cholera challenge trials. These models also helped evaluate duration of protection and guided down-selection of vaccine candidates. These models differ from influenza challenge trials in several significant ways: study participants did not have pre-existing immunity, the route of administration was identical to CAI, and there was less risk of community exposure. But several features of the cholera and typhoid vaccine challenge models were key to their success: well-characterized challenge pathogens, effective rescue therapy for mitigating risk to participants, and standardization of clinical endpoints.

### 1.2 Industry perspective on value of CHIVIM

Industry experts agreed that CHIVIM could become a useful tool in the development of commercially-produced vaccines, especially if the model can assess the potential for broader protection. The model could also be effective for validating products that have new mechanisms of action, and for products that lack known CoPs. In the industry research pipeline, CHIVIM may be most useful for deciding whether or not to proceed with larger studies, rather than for down-selection between various candidate vaccines. CHIVIM studies could be conducted pre- and post-licensure as new and diverse challenge viruses become available to extend the evidence for broad protection.

There was agreement that the model must be well-developed for industry needs and be predictive for prevention of clinically meaningful disease. The pathway to licensure of novel influenza vaccines will require demonstration that vaccination reduces the risk of disease, and the necessary trials will have to be large and conducted over several years to accrue sufficient evidence of broad and durable protection. Manufacturers will need to manage the risk of making these development investments by generating pre-clinical and early-stage clinical evidence supporting the effectiveness of their vaccine candidate. Experts noted that challenge virus stocks could have limited lifespans, and that investment of time and expense for the production of challenge viruses without assured market potential could be an issue. There was also concern about the risk for discontinuation of a product development program that is potentially viable against natural infection because of poor performance in a CHIVIM study.

For industry, CHIVIM studies may play a role in the universal vaccine development process but must demonstrate value that is comparable to the time and investment required. A clear pathway to licensure for a potential universal influenza vaccine is needed in order for companies to be willing to invest in CHIVIM.

## 2 Topic 2: Regulatory and ethical implications of the CHIVIM

Regulatory experts agreed that well-characterized human influenza challenge models could contribute data that support regulatory approval of broadly protective influenza vaccine candidates. However, there is currently no consensus among regulatory agencies about the role of challenge models in vaccine approval pathways. There was agreement that the WHO should create harmonized guidance for influenza challenge trials intended to support universal vaccine approval, with input from the WHO Product Development for Vaccines Advisory Committee.

Regulatory experts agreed that HCTs are best for evaluating efficacy (rather than safety) of candidate vaccines and could be an efficient way to select candidate vaccines for field trials. There was agreement that CHIVIM may be most useful for understanding immune CoPs and other markers, and for evaluating efficacy of vaccines intended to attenuate severity/length of disease by activating cellular immunity. It was noted that for CHIVIM to be effective in this role, it should induce illness with adequate intensity/duration that is not confounded by post-challenge antiviral therapy. In addition, the term “universal vaccine” is unlikely to appear in a product label, but challenge trials could provide evidence of broader protection against influenza that could be included in the prescribing information. Finally, CHIVIM studies alone are not likely to produce sufficient evidence of clinical benefit to support an accelerated product approval based on the current regulatory approaches.

With regards to the ethics of human challenge trials, experts emphasized the importance of weighing any potential public health benefits against the risks associated with CHIVIM. There was agreement that more guidance is needed in this area, particularly with regards to assessing environmental risk. A proposed Zika HCT was not approved due to concerns about subject risk versus social benefit, and especially potential third-party risk [14]. Challenge trials with wild type human influenza viruses have been approved in the US and other countries [9], [11], [15], and studies with low pathogenic avian influenza viruses (LPAIs) are being considered. Guidance about risk benefit analysis could be developed based on previous trials and best practices for protecting against third-party risk.

US regulations concerning Dual Use Research of Concern (DURC)[16] and the policy for Potential Pandemic Pathogen Care and Oversight (P3CO)[17] were discussed with regards to oversight of production and investigational use of historical seasonal influenza strains. A majority of panelists and participants agreed that avian strains may fall under these regulations, whereas historical seasonal strains likely do not. However, older historical strains for which there is little or no remaining population immunity may also present risk to the community.

All agreed that identical ethical standards for HCTs should maintained when conducting research in low and middle-income countries (LMICs). Subject compensation can vary by location, as long as payments are non-coercive and study participants fully understand potential risk.

## 3 Topic 3: Selection of challenge virus strains

In order for CHIVIM to address multiple research questions, a full panel of well characterized challenge viruses is needed. The desired preclinical and clinical criteria for human influenza challenge viruses should be established, as well as consensus on preferred sources and production substrates (eggs, cell cultures). There are several important preclinical criteria for challenge viruses. They should be derived from an acceptable source (clinical isolate recovered in a suitable substrate, isolated from well-characterized subject, reverse genetics (RG)), and should be produced in a suitable production substrate and according to current Good Manufacturing Practice (GMP). They should exhibit genetic stability, and there should be no evidence of adventitious agents. Finally, challenge viruses should be susceptible to available antiviral therapies. Clinically, challenge viruses should induce moderate, overtly symptomatic, uncomplicated disease that can be confirmed by detection of influenza virus replication, with complete recovery in healthy participants. Safety, established through trials, should be an important criterion for virus selection.

Currently there are several H1N1 as well as H3N2 challenge strains that have been used in recent studies. (Table 2) Additional challenge strains are needed to address research questions about pathogenesis, transmission, and CoPs, The panel discussed alternative sources of challenge virus for particular circumstances. For children and other high-risk groups, licensed live attenuated influenza vaccine (LAIV) could be an alternative to wild type seasonal virus challenge. Avian LAIV candidates, LPAI strains, or historic seasonal viruses could be used to assess breadth of vaccine protection, though avian LAIVs and LPAI viruses generally produce limited or no symptoms in adults and minimal, inconsistent viral shedding.

**Table 2:** Challenge Agents From Recent Studies.

	Virus	Source	Substrate
H1N1	A/New Caledonia/99		
	A/Brisbane/59/2007	Unknown	Eggs
	A/California/04/2009 also known as A(H1N1)pdm09	Reverse genetics	Vero cells
	A/California/04/2009-like	Patient sample	Eggs

H3N2	A/Wisconsin/67/2005	Patient sample	1°CK × 3, Egg × 4, Vero × 2
	A/Perth/16/2009	Reference virus	Eggs
	A/Belgium/4217/2015	Patient sample	MDCK cell - Eggs
	A/Bethesda/MM1[15]	Reverse genetics	Vero cells

It was suggested that multiple strains of the same influenza A subtype may be needed in order to adequately challenge a broadly protective vaccine. If a candidate vaccine elicits antibodies that target the HA “stem” region of H1N1 or H3N2 and is found to be protective, demonstration of similar anti-HA stem responses to other influenza A subtype viruses in the same phylogenetic group by *in vitro* testing would support an inference of broad protection.

### 3.1 Recreating historical strains

Using contemporary influenza virus strains as challenge agents is the most feasible approach from a risk management perspective, but many study participants would have immunity resulting in low infectivity. However, younger adult participants should be more susceptible to historical strains that are no longer circulating, which could facilitate subject recruitment, increase attack rate, and induce more overt illness and/or greater virus shedding. Historical strains may serve as a better test for broadly protective candidate vaccines and should have a longer period of viable use (compared to contemporary seasonal strains). Historical antigenic drift isolates that emerged but failed to persist were suggested as an attractive subset of historical strains to be considered. Regardless, the risk of historical strains escaping into the community is of concern, and the value of available influenza vaccines to protect staff or contain a community outbreak may be limited.

Due to cross-reactivity between current circulating strains and many historical H1N1 strains, young adults aged 18–30 should have increased susceptibility to H1N1 viruses that circulated exclusively between 1977 and 1984. RG could be used to derive the most promising strains from that period, using wild type and A(H1N1)pdm09 variants as backbone, and potentially including a suicide mechanism. A historical approach to define antigenically distant H3N2 and influenza B challenge agents could be used as well.

There was noted consensus among the panelists that H2 influenza strains, though possibly well-suited for evaluating pandemic vaccine candidates for immunogenicity or for protection in animal models, should not be used as a challenge agent due to the risk of re-introduction into the mostly naïve (those born after 1968) community.

### 3.2 Managing community risk from historical challenge strains

There are existing tools that could be adapted for assessing the safety of using historical strains as challenge agents for candidate universal vaccines. The Centers for Disease Control and Prevention Influenza Risk Assessment Tool (IRAT)[18] evaluates risk from emerging zoonotic influenza strains in two areas, likelihood of emergence and likelihood of impact, by weighting various predetermined risk elements. The strain with the highest risk for emergence/impact currently is H7N9. This tool could be adapted to evaluate the risk of using historical or novel challenge strains by selecting appropriate risk elements.

The WHO Tool for Influenza Pandemic Risk Assessment (TIPRA)[19] could also be useful for assessing challenge strains. TIPRA takes into consideration three groups of characteristics: the properties of the virus (genomic characteristics, susceptibility to antivirals, etc.), its attributes in human population (disease severity, population immunity, etc.) and the virus ecology and epidemiology in non-human hosts. Perhaps with some modifications, this tool could be useful for comparing the relative risk associated with different historical strains.

The panel discussed potential strategies for producing RG challenge viruses with safety features (sensitivity to amantadine and oseltamivir, truncated NS1 proteins, temperature sensitivity, etc.) that could mitigate risk to the community should the virus escape containment.

The WHO has guidelines for clinical trial design that can help to reduce environmental risk, such as using personal protective equipment (PPE) [20]. Strain-appropriate containment strategies should be adopted. Some trials may require quarantine. Conducting trials outside of influenza season may also help lower risk to community.

### 3.3 The role of WHO Collaborating Centers

The WHO Global Influenza Surveillance and Response System (GISRS) is a network of research centers and laboratories in more than 100 countries that work together to sample and evaluate circulating influenza virus strains. Six WHO Collaborating Centers (CCs) isolate and sequence new influenza variants, as well as perform detailed antigenic analysis and antiviral sensitivity testing. Candidate vaccine viruses are propagated in eggs and in qualified cell lines (following standard operating procedures (SOPs)), and the list of available viruses is updated every six months. Adventitious testing is the responsibility of vaccine manufacturers. These WHO CCs are therefore well situated to perform a similar role for the selection of candidate challenge viruses.

The need for an available cell line for production of challenge viruses and the lack of necessary reagents was discussed. Once a process for strain selection is established, it is recommended that a government agency such as the National Institute of Allergy and Infectious Diseases (NIAID) or non-profit produce the challenge viruses in a dedicated cell line, using standardized reagents and protocols. NIAID is currently funding the production of H1N1 and H3N2 challenge strains, and has agreed to make them available to the extramural research community. Challenge strains produced in the US could be used in other countries, although a Material Transfer Agreement may be required in some cases. Extensive adventitious testing of any potential challenge strain would be necessary.

## 4 Topic 4: Increasing standardization, access, and capacity

### 4.1 CHIVIM standardization

Standardization of CHIVIM assays and clinical protocols is needed for reliability and comparison of results between researchers and institutions. Some approaches to improving standardization of assays between research laboratories include assay harmonization, use of biological standards, proficiency testing of labs, and centralized testing. Studies on inter-laboratory variability using harmonized assay protocols have shown mixed results, although detailed protocols with specific parameters are more useful than general recommendations [21]. The use of biologic standards (such pooled sera as standard for influenza testing) can reduce the amount of variation between labs [22]. Sharing standardized reagents decreases variability between labs, and biologic standards can be also used to decrease variability of results when harmonized assays are not available.

Harmonized study protocols have been successfully developed and implemented for clinical trials. One issue for influenza vaccine challenge trials is the need for comparator vaccines. As the susceptibility of any study cohort to a particular challenge virus is likely to vary from trial to trial, using a licensed seasonal influenza vaccine as a control treatment in addition to a placebo-treated group would provide useful context by enabling an assessment of a vaccine candidate’s efficacy relative to the current standard of care as well as relative to no vaccination.

There are several consortia that support collaboration between researchers and institutions working on influenza vaccines. The CONSISE consortium [23] is a global network for standardization of the seroepidemiology of influenza and other respiratory pathogens. The FLUCOP Project [24] works to improve standardization of HI and virus neutralization (VN) assays for CoP studies in seasonal influenza vaccine trials. UNISEC [25] is a consortium of academic researchers, public health institutes, and industry members from seven European countries and Israel which was created to share influenza vaccine expertise. ISARIC [26] is a standardized data collection tool for severe acute respiratory infections which was developed in collaboration with WHO. These platforms serve as repositories for shared protocols, harmonized assays, SOPs, and clinical trial networks. Knowledge from these consortia may be applicable to the further development and standardization of CHIVIM. However, CHIVIM may require a consortium of its own, to facilitate effective sharing of information and resources between institutions. The potential success of a CHIVIM consortium would depend on the involvement of major stakeholders (including industry), enthusiasm of participating researchers, and good administrative support, and may require some time to become established.

### 4.2 Access to CHIVIM

The four institutions that are currently conducting CHIVIM studies (WCCT Global, hVIVO, SGS Belgium NV, and NIAID) produce their own challenge virus strains using specific manufacturing and quality controls. In order to create more access to challenge viruses and improve standardization, a common repository of challenge viruses is needed. WHO could provide guidance on strain selection, as discussed above.

### 4.3 Building capacity

The need for more CHIVIM studies will depend on the number of candidate vaccines in development. Standardization of the model and access to materials will facilitate the creation of new study centers to increase capacity as needed. Although influenza challenge trials are currently limited to European countries and the US, there may be advantages to conducting these trials in LMICs as well, particularly in places with increased genetic diversity and/or less established immunity from routine influenza vaccination, and to build vaccine development capacity in low-resource communities where the burden of influenza may be most profound.

When conducting studies in LMICs, there are some additional factors to consider, such as the time necessary to achieve adequate community engagement, informed consent and appropriate compensation, infrastructure limitations and ensuring adequate containment of challenge virus, and whether the investigational product will become accessible to the population.

## 5 The path forward: Steps needed to develop CHIVIM for universal influenza vaccine research

Goal: Universal influenza vaccine licensure

The ultimate goal for CHIVIM is to accelerate the accumulation of knowledge needed to develop and license broadly protective influenza vaccines offering long lasting protection against both seasonal and pandemic disease.

(1) Standardize the model

The issue of standardization was a recurring theme during the discussions, particularly with regard to the effectiveness of CHIVIM as a tool for vaccine development. Standardization of the model is critical to produce meaningful, comparable results from CHIVIM trials that can support universal vaccine licensure. The list of CHIVIM components that require standardization is extensive and includes challenge virus production and release procedures, reagents, trial protocols, assays, route of challenge administration and delivery methods, rescue protocols, safety protocols, and comparator vaccines. WHO and other key stakeholders including regulatory agencies and industry should collaborate to develop harmonized guidance on the standardization needed to make CHIVIM a more precise tool.

(2) Manufacturing of challenge virus stocks

Selecting and producing appropriate challenge viruses may be the rate-limiting step in the realization of CHIVIM. Currently there is a limited number of available strains and lack of guidance for the selection and production of challenge virus. Challenge viruses must be well-characterized and matched to the research questions being investigated. To this end, the recommendation is for WHO to take the lead on the selection of strains and evaluation of seed stocks, taking into consideration the desired preclinical and clinical characteristics for influenza challenge discussed above. WHO should also provide guidance on GMPs that are specific to influenza challenge virus strains.

Special consideration and consensus are needed for the issue of using historical influenza strains, viruses produced with reverse genetics, LPAI strains, or other controversial challenge viruses to address particular research questions.

All well-characterized influenza virus challenge agents should be made available to qualified institutions for CHIVIM trials.

(3) Mitigate Risk

Experts agreed that safety should be the primary consideration when designing CHIVIM trials. A WHO white paper addressing the assessment and mitigation of risks associated with CHIVIM is needed. Risk mitigation strategies should be standardized and address all potential risks to study participants, staff, and community.

(4) Create a research network and refine the objective measures of influenza disease

A research consortium should be created for institutions conducting CHIVIM trials for universal vaccine development, allowing for the free exchange of resources, best practices, and data between participating members. Challenge viruses, harmonized protocols, and standardized assays should be made available to all CHIVIM study sites. As the clinical syndrome following influenza virus challenge is anticipated to be mild, refined objective measures of illness and altered airway function should be devised to assess the benefit of vaccination.

(5) Expand Access and Capacity

The number of research centers performing challenge studies for universal vaccine development should increase. The research consortium network and standardization of the model will facilitate an increase in capacity. Expansion to LMICs is recommended where feasible, with special attention to ensuring adequate containment, community involvement, and informed consent.

(6) Assess Results and Further Develop CHIVIM as Needed

As these steps are taken and the results from CHIVIM studies become available, further refinement of the model may be necessary. Novel vaccine mechanisms, further understanding of immune response, or other new information may provoke research questions that will require modifications to the model.

All authors attest they meet the ICMJE criteria for authorship.
